# Docetaxel induces moderate ovarian toxicity in mice, primarily affecting granulosa cells of early growing follicles

**DOI:** 10.1093/molehr/gau057

**Published:** 2014-07-30

**Authors:** Federica Lopes, Rowena Smith, Richard A. Anderson, Norah Spears

**Affiliations:** 1 Centre for Integrative Physiology, University of Edinburgh, Edinburgh EH8 9XD, UK; 2 MRC Centre for Reproductive Health, University of Edinburgh, Edinburgh EH16 4TJ, UK

**Keywords:** apoptosis, docetaxel, mouse, ovarian follicles, thyroid hormone

## Abstract

Advances in cancer therapy have focused attention on the quality of life of cancer survivors. Since infertility is a major concern following chemotherapy, it is important to characterize the drug-specific damage to the reproductive system to help find appropriate protective strategies. This study investigates the damage on neonatal mouse ovary maintained *in vitro* for 6 days, and exposed for 24 h (on Day 2) to clinically relevant doses of Docetaxel (DOC; low: 0.1 µM, mid: 1 µM, high: 10 µM). Furthermore, the study explores the putative protective action exerted by Tri-iodothyronine (T3; 10^−7^ M). At the end of culture, morphological analyses and follicle counts showed that DOC negatively impacts on early growing follicles, decreasing primary follicle number and severely affecting health at the transitional and primary stages. Poor follicle health was mainly due to effects on granulosa cells, indicating that the effects of DOC on oocytes were likely to be secondary to granulosa cell damage. DOC damages growing follicles specifically, with no direct effect on the primordial follicle reserve. Immunostaining and western blotting showed that DOC induces activation of intrinsic, type II apoptosis in ovarian somatic cells; increasing the levels of cleaved caspase 3, cleaved caspase 8, Bax and cleaved poly(ADP-ribose) polymerase, while also inducing movement of cytochrome C from mitochondria into the cytosol. T3 did not prevent the damage induced by the low dose of DOC. These results demonstrated that DOC induces a gonadotoxic effect on the mouse ovary through induction of somatic cell apoptosis, with no evidence of direct effects on the oocyte, and that the damaging effect is not mitigated by T3.

## Introduction

As a consequence of advances in modern cancer care, an increasing number of women are long-term survivors following treatment. Nevertheless, in many cases, patients undergoing intensive treatment experience severe side-effects, either immediately or even years after the therapy. It is well recognized that some anticancer drugs are gonadotoxic, with infertility now representing a major concern for women undergoing cancer treatments, particularly so for younger patients (Meirow *et al.*, 2010). The key basis for the adverse effects on the ovary is the limited supply of oocytes contained within the ovarian follicles, which are formed before birth. This population of primordial follicles represents a female's ovarian reserve. The reproductive lifespan of a woman ceases when the number of primordial follicles becomes too low (less than around a thousand), leading to the menopause (Wallace and Kelsey, 2010). Insults and pathological circumstances affecting the ovary can precociously deplete this reserve, leading to premature ovarian insufficiency (POI) with loss of fertility and the consequences of estrogen deficiency.

The severity of ovarian toxicity caused by the taxane class of chemotherapeutic agents has been difficult to determine, in part because they are usually administered in association with other drugs and other adjuvants (Han *et al.*, 2009; Pérez-Fidalgo *et al.*, 2010; Torino *et al.*, 2012). Docetaxel (DOC), a second generation taxane, represents one of the most powerful chemotherapeutic drugs against local or metastatic breast, non-small cell lung and ovarian cancer and displays a high level of radio-sensitizing activity (Gligorov and Lotz, 2004). DOC triggers cells into a programmed cell death pathway, mainly by binding with the β-subunit of tubulin, thus stabilizing microtubules and inhibiting their depolymerization. This results in cell cycle arrest, followed by phosphorylation of bcl-2 and apoptosis (Subrata *et al.*, 1997). Taxanes can also directly change mitochondrial membrane permeability, ultimately activating caspases (Fabbri *et al.*, 2006).

DOC plasma concentrations in patients are highly variable, with different regimens of 40 mg m^−2^ administered weekly or 100 mg m^−2^ administered every 3 weeks used clinically. Pharmacokinetic studies report a plasma concentration of 0.1–1.2 µM and maximum concentrations (*C*_max_) ranging between 0.3 and 6.9 µM within 24 h post-treatment (Baker *et al.*, 2004; Slaviero *et al.*, 2004; Brunsvig *et al.*, 2007).

There is some evidence from clinical studies that DOC increases reproductive impairment when administered in combination with other drugs (Martin *et al.*, 2005; Han *et al.*, 2009) and there is evidence in an animal model of loss of primordial follicles after treatment with the related drug paclitaxel (Gucer *et al.*, 2001). However, effects of DOC are particularly controversial, with other work showing that patients treated with taxanes restore normal menstrual cyclicity more frequently than those treated with other drugs (Minisini *et al.*, 2009; Zhou *et al.*, 2010). Ovarian damage has been difficult to assess directly, and most studies have used chemotherapy-induced amenorrhoea as a surrogate for POI, a method that does not allow detection of incomplete ovarian damage. Using a detailed assessment of ovarian function, we previously reported evidence for taxane-induced toxicity in women treated for early breast cancer (Anderson *et al.*, 2006).

It is likely that each anticancer drug will affect ovarian tissue differently, according to its mechanism of action (Morgan *et al.*, 2012). Drug-specific damage therefore needs to be characterized to define toxicity, with the aim of then finding protective approaches. One possible protective strategy could be to target the ovary with a compound that antagonizes chemotherapy toxicity, but so far this approach has been explored only by laboratory studies. Such studies have investigated different molecular approaches, such as sphingosine-1-phosphate, the retrovirus-mediated multidrug resistance gene and the c-Abl inhibitor imatinib (Morita *et al.*, 2000; Gonfloni *et al.*, 2009; Salih, 2011; Morgan *et al.*, 2012). As one example of this kind of protective approach, thyroid hormones have been hypothesized to minimize the impact of taxanes on ovarian functionality, due to their role on cell proliferation and apoptosis in ovarian somatic cells (Verga Falzacappa *et al.*, 2009, 2012). To date, one report has shown that 3,5,3′-triiodothyronine (T3) is able to counteract the apoptosis of rat ovarian GCs exposed to the taxane paclitaxel (Verga Falzacappa *et al.*, 2012).

Here, a culture system of neonatal mouse ovaries has been used to investigate DOC-induced damage to the resting pool of primordial follicles and to the early stages of follicular development. This *in vitro* system has been demonstrated to be a reliable and physiological model for studying effects of compounds on folliculogenesis in a highly controlled environment (Morgan *et al.*, 2013). In addition, in order to explore potential protective strategies, the role of T3 supplementation has been investigated for its ability to prevent DOC-induced damage in the intact ovary.

## Materials and Methods

### Animals

All experiments were approved by the University of Edinburgh's Local Ethical Review Committee and were carried out in accordance with UK Home Office regulations. C57Bl/J6 mice were housed in a 14 h light-10 h dark cycle, with food and beverage provided *ad libitum*.

### Tissue culture

Newborn mouse ovary culture was performed according to Morgan *et al.* (2013). In brief, on the day of birth, female mice were sacrificed by decapitation and ovaries dissected out and placed in pre-warmed Leibovitz L-15 medium (Invitrogen, UK) supplemented with 3 mg ml^−1^ bovine serum albumin (BSA; Sigma-Aldrich Ltd, UK) and kept at 37°C. Whole ovaries were transferred into a 24-well plate (Grenier Bio-one, Stonehouse, UK) containing α-Minimum Essential Medium (Invitrogen, UK) supplemented ith 3 mg ml^−1^ BSA, placed on floating polycarbonate membranes (Whatman Nucleopore Polycarbonate Membrane, Camlab Ltd, Cambridge, UK) and incubated in a 5%CO_2_ atmosphere at 37°C (Day 1). On the second day of culture (Day 2), medium was supplemented with 0.1% ethanol-vehicle (CONTROL) or with one of three Docetaxel concentrations (Sigma-Aldrich Ltd, UK): 0.1 µM (LOW-DOC), 1 µM (MID-DOC) or 10 µM (HIGH-DOC). Doses of DOC were chosen to cover the range of concentrations found in the plasma of patients undergoing cancer treatments (Baker *et al.*, 2004; Brunsvig *et al.*, 2007). On Day 3, all ovaries were transferred into fresh control medium. Half of the medium was replaced on Day 5, with the culture continued to the end of Day 6. Previous work using this experimental paradigm has shown that this time frame supports the health of control cultures, and allows growth initiation of the majority of follicles (Morgan *et al.*, 2013). Intracellular location of cytochrome C was examined in control and HIGH-DOC treated ovaries. In each run of the cytochrome C experiment, four ovaries were dissected from mice, two placed in CONTROL and two in HIGH-DOC conditions. After 6 days in culture, pairs of ovaries were processed for western blotting as detailed below.

In the T3 experiment, ovaries were cultured as above but exposed on Day 2 to: 0.1% ethanol-vehicle (CONTROL), 10^−7^ M of T3 (T3), 0.1 µM of DOC (LOW-DOC) or a combination of 10^−7^ M of T3 and 0.1 µM DOC (T3+LOW-DOC). T3 was supplied by Sigma-Aldrich Ltd (UK). The lower DOC dose was chosen because it resulted in a robust increase in the percentage of unhealthy follicles but with less extensive damage than the two higher doses. The concentration of 10^−7^ M T3 was chosen on the basis of a previous paper showing that this concentration of T3 is able to overcome paclitaxel-induced damage to GCs (Verga Falzacappa *et al.*, 2012; Verga Falzacappa, personal communication). Ovaries in the T3 and T3+LOW-DOC groups were also exposed to T3 during Days 3–5, but not to DOC after Day 2. The culture finished at the end of Day 6. For each experiment, ovaries were processed at the end of the culture period for histology, western blotting or immunohistochemistry (IHC), as detailed below.

### Follicle classification and quantification

On Day 6 of culture, ovaries were fixed in Bouin's fluid and embedded in paraffin wax. Serial sections (5 µm thick) were collected and every sixth section stained with haematoxylin and eosin and photomicrographed (DMLB Leica microscope, Leica Microsystem Ltd, UK). Follicle counts were performed with the assessor blind to treatments and using ImageJ software. Total number of follicles per ovary was estimated from counts of follicles with a visible germinal vesicle in the section, with counts then corrected using the Abercrombie formula (Abercrombie, 1946). Follicles were classified in accordance with Morgan *et al.* (2013). Follicles were considered as primordial when only flattened pre-granulosa cells (GCs) were present, as transitional when some cuboidal GCs were mixed with flat pre-GCs and at the primary stage when a uniform layer of cuboidal GCs was present. Follicle health was assessed using standard morphological criteria: follicles were judged as unhealthy if they had (i) an oocyte with eosinophilic, shrunken or non-homogeneous cytoplasm, or condensed nuclear chromatin; (ii) GCs with condensed chromatin or irregular shapes; (iii) unhealthy oocyte and GCs.

### Immunohistochemistry

At the end of the culture, ovaries were fixed in 10% neutral buffered formalin (Sigma-Aldrich Ltd, UK) overnight at 4°C, wax embedded and serial sectioned at 5 µm. IHC for cleaved caspase 3 (CC3) and mouse vasa homologue (MVH) used fluorescent secondary antibodies, while IHC for cleaved caspase 8 (CC8) used an avidin-biotin visualization system.

#### CC3/MVH

Every 12th section was de-waxed and rehydrated through a graded series of ethanol and water. Slides were microwaved in 10 mM sodium citrate (pH6; Fisher Chemical, Loughborough, UK) and blocked in 20% normal goat serum in phosphate-buffered saline (PBS; Fisher Scientific UK Ltd, UK) with 0.1% Triton X-100 (PBST), and 5% BSA. Slides were then incubated overnight at 4°C with rabbit anti-CC3 (1:500; Cell Signalling Technology, USA) and mouse anti-MVH (1:100; Abcam, UK) antibodies. After washings in PBST, slides were incubated for 1 h at room temperature with secondary antibodies: Alexa Fluor 568 goat anti-mouse IgG_1_ (1:200; Invitrogen, UK) and goat anti-rabbit biotinylated (1:200; DakoCytomation, Denmark), followed by 30 min at room temperature with Alexa Fluor 488 streptavidin conjugate (1:200; Invitrogen, UK). After washes in PBST, slides were counterstained with 4′,6-Diamidino-2-phenylindole (DAPI; Invitrogen, UK) and mounted in Vectashield mounting medium (Vector Laboratories, USA). Fluorescent images were taken with a Leica DM5500B microscope on a DFC360FX camera. Image analysis was performed with ImageJ software, with the assessor blind to treatments. Follicles were counted only when a MVH-positive oocyte with a DAPI-positive nucleus was visible in the section. In those instances, apoptotic (CC3-positive) GCs and/or oocyte were recorded. The degree to which apoptotic cells were present in the ovaries was also assessed by measuring the area of CC3 stained cells in the section relative to the area (DAPI) of the section.

#### CC8

One or two middle sections per ovary were de-waxed and rehydrated through a graded series of ethanol and water. Endogenous peroxidase was blocked in 3% H_2_O_2_ and 10% methanol (Fisher Chemical, Loughborough, UK) in PBS, then microwaved in 10 mM sodium citrate (pH6; Fisher Chemical, Loughborough, UK) and blocked in 20% normal goat serum in PBST, and 5% BSA. Slides were then incubated overnight at 4°C with rabbit monoclonal antibody anti-CC8 (1:500; Cell Signalling Technology, USA). After washing in PBST, slides were incubated for 1 h at room temperature with a biotinylated secondary goat anti-rabbit (1:200; DakoCytomation, Denmark), re-washed in PBST, incubated with avidin and biotinylated horse-radish peroxidase (Vectastain Elite ABC Kit, Vector Laboratories, USA) for 30 min then visualized by staining with 3, 3′-diaminobenzidine (DAB, Vector Laboratories, USA), counterstained with haematoxylin and photomicrographed (DMLB Leica microscope, Leica Microsystem Ltd, UK). Image analysis was performed with ImageJ software, with the assessor blind to treatments. The degree to which apoptotic cells were present in the ovaries was assessed by measuring the area of DAB-positive (CC8-expressing) stained cells in the section, relative to section area.

### Polyacrylamide gel electrophoresis and western blotting

On Day 6 of culture, ovaries were washed in ice-cold PBS and snap frozen on dry-ice. Twenty micro-litres of lysis buffer containing 50 mM HEPES, 50 mM NaCl, 1% Triton X-100, Phosphatase Inhibitor Cocktails 2 and 3 (all from Sigma-Aldrich Ltd, UK) and Complete protease inhibitors cocktail (Roche Diagnostic Ltd, UK), were added to two ovaries, then homogenized and centrifuged. Ovaries (*n* = 20), after freezing, underwent a process for the separation of mitochondria and cytosol components (Mitochondria Isolation kit for cultured cells, Thermo Scientific, Rockford, USA) with a few modifications to the manufacturer's instruction. In both cases, 10 µg protein was loaded onto a NuPAGE^®^ Novex^®^ 4–12% Bis-Tris pre-cast polyacrylamide gel (Invitrogen, UK). Following electrophoresis, proteins were then transferred onto a nitrocellulose membrane, and membranes probed overnight at 4°C with 1:1000 anti-PARP (poly(ADP-ribose) polymerase) rabbit polyclonal antibody (New England Biolabs, Hertfordshire, UK), 1:400 rabbit monoclonal antibody to Bcl2-associated X protein (Bax;Abcam, Cambridge, UK), or 1:1000 rabbit monoclonal antibody anti-cytochrome C (Abcam, Cambridge, UK) and 1:2500 rabbit polyclonal antibody to β-actin (as internal standard) (Abcam). After washing in 0.1% Tween20 (Fisher Chemical, Loughborough, UK) in PBS, blots were incubated with Alexa Fluor 750 goat anti-rabbit (1:5000; Invitrogen, UK) for 1 h at room temperature. Fluorescence was detected by Li-cor scanner with Odyssey v1.2 software (Li-cor Biosciences, USA).

### Statistical analysis

All data were analysed using GraphPad Prism software. Normal distribution was assessed using a non-parametric test of distribution; the Kolmogorov–Smirnov test. To determine statistical significance between the control group and more than one treatment group, one-way ANOVA followed by Bonferroni *post hoc* tests were applied to normally distributed data, while Kruskal–Wallis followed by Dunn *post hoc* tests were applied to data that were not normally distributed. Where controls were compared with a single treatment, a paired *t*-test was used. All results are presented as mean ± SEM. Statistical significance was set at values of *P* < 0.05.

## Results

### Docetaxel affects the number of follicles that reach the primary stage

**Figure 1:**
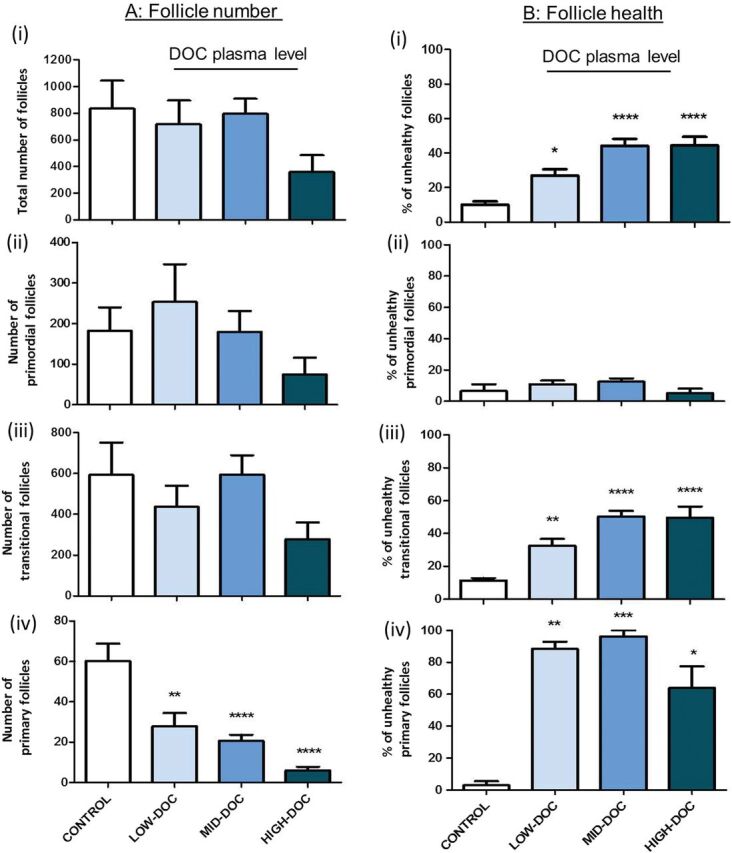
Docetaxel affects the number and health of early growing but not primordial follicles in mice. (**A**) Follicle number. Whole mouse ovaries were maintained *in vitro* for 6 days and treated with Docetaxel (DOC) for 24 h on Day 2 at concentrations of 0.1 µM (LOW-DOC), 1 µM (MID-DOC) or 10 µM (HIGH-DOC). The data show that DOC significantly decreased the number of primary follicles only. (**B**) Follicle health. DOC had significant effects on health in all but the primordial follicles. Total of ovaries used in three independent experiments: CONTROL = 7, LOW-DOC = 7, MID-DOC = 8 and HIGH-DOC = 7. One-way analysis of variance (ANOVA) followed by Bonferroni *post hoc* tests were applied. Data are mean ± SEM. *P* < 0.05 (*), *P* < 0.01 (**), *P* < 0.001 (***), *P* < 0.0001 (****) for DOC versus CONTROL.

Newborn mouse ovaries were randomly allocated to one the following experimental groups: CONTROL (*n* = 7), LOW-DOC (*n* = 7), MID-DOC (*n* = 8) or HIGH-DOC (*n* = 7). Exposure of ovaries to DOC led to a specific reduction in the number of primary follicles (Fig. 1A) without a change in the overall follicle number. The number of primary follicles was significantly reduced when ovaries were exposed to DOC in a dose-dependent fashion (Fig. 1A-iv). The total follicles number per ovary did not vary at LOW-DOC and MID-DOC, while a marked decrease was observed at HIGH-DOC compared with the number of follicles in the CONTROL group, but this was not statistically significant (*P* = 0.07; Fig. 1A-i). Similarly, the number of primordial and transitional follicles did not decrease significantly when exposed to DOC (Fig. 1A-ii and -iii). 

### Docetaxel damages follicle health

Follicle health was affected more markedly then follicle number (Fig. 1B). In fact, despite the relatively good preservation of the total ovarian follicle population, a dramatic decrease in follicle quality was observed, with a significant increase in the percentage of unhealthy follicles even after exposure to the lowest concentration of DOC (Fig. 1B-i). When unhealthy follicles were further categorized within each developmental stage, primordial follicles did not show signs of damage (Fig. 1A-ii). In contrast, growing (transitional and primary) follicles were greatly affected by DOC treatment, with a significant increase in the percentage of unhealthy follicles observed (Fig. 1B-iii and -iv). Although there was a lower percentage of unhealthy primary follicles in the HIGH-DOC than in the LOW- and MID-DOC groups, this may reflect the marked depletion of that follicle stage, as only 5 ± 1 primary follicles were left in these ovaries (Fig. 1A-iv and B-iv).

### Docetaxel primarily affects GCs of early growing follicles

**Figure 2:**
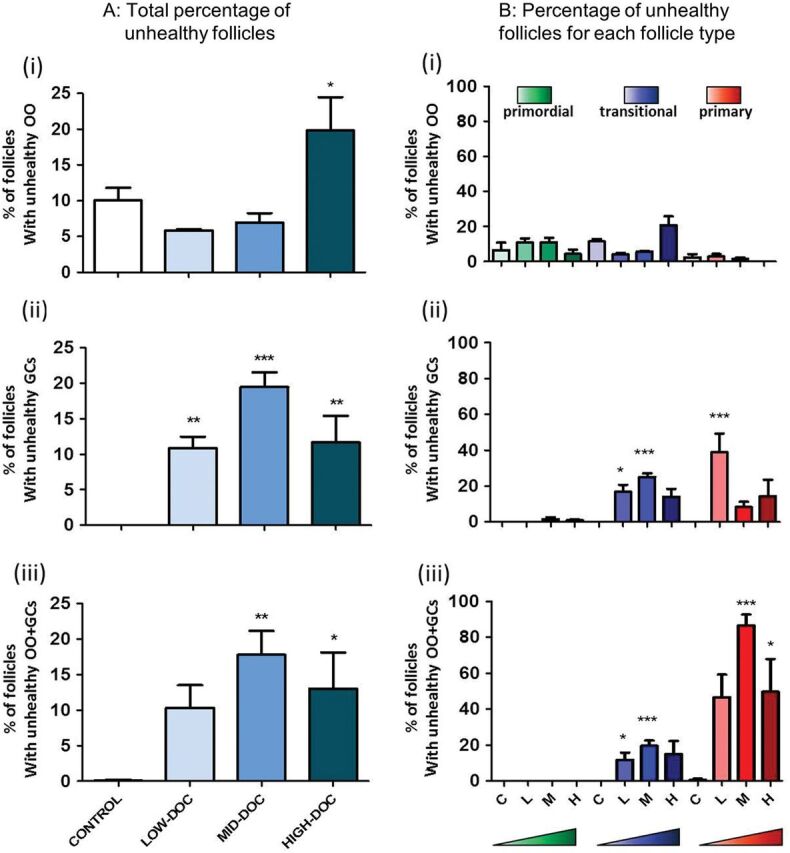
Docetaxel primarily damages GCs of growing follicles in mice. All unhealthy follicles were further classified as being unhealthy due to the presence of (i) unhealthy oocyte only; (ii) unhealthy GCs only; or (iii) both unhealthy oocyte and GCs (OO+GCs). (**A**) Total percentage of unhealthy follicles. An effect on health was seen in the follicles with unhealthy GCs only, where even LOW-DOC induced a significant increase: in contrast, only the HIGH-DOC treatment significantly increased the percentage of follicles with unhealthy oocytes. (**B**) Percentage of unhealthy follicles for each follicle type. Primordial follicles were unaffected by drug treatment, but growing transitional and primary follicles both exhibited poorer follicle health, with an increase in the percentage of follicles with unhealthy GCs only and in the OO+GC category, after exposure to DOC. CONTROL = C, LOW-DOC = L, MID-DOC = M, HIGH-DOC = H. Triangles and stronger colours indicate increasing DOC doses. Total of ovaries used in three independent experiments: CONTROL = 7, LOW-DOC = 7, MID-DOC = 8 and HIGH-DOC = 7. One-way ANOVA followed by Bonferroni *post hoc* tests were applied. Data are mean ± SEM. *P* < 0.05 (*), *P* < 0.01 (**), *P* < 0.001 (***) for DOC versus CONTROL.

**Figure 3:**
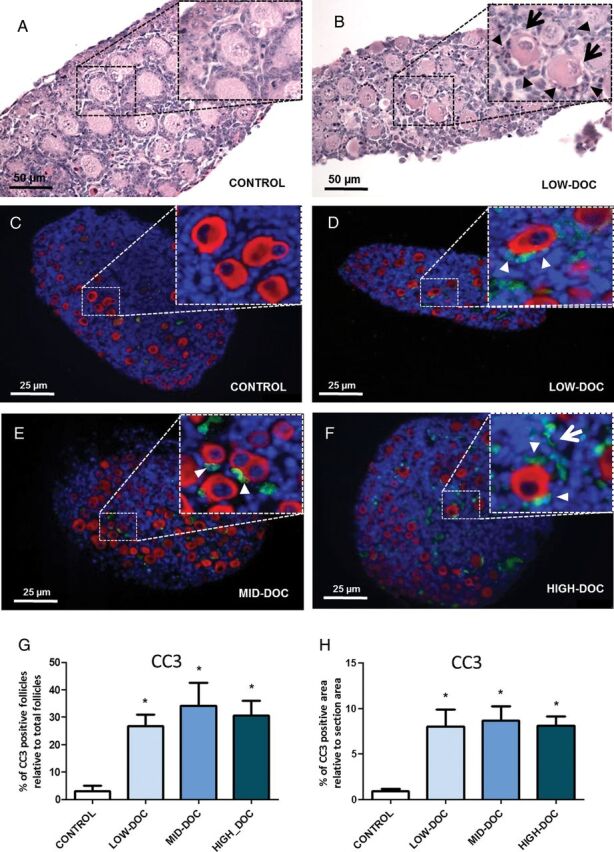
Docetaxel induces ovarian damage in mice activating caspase 3 in ovarian follicles and stroma. (**A** and **B**) Representative photomicrographs of mouse ovarian sections stained with haematoxylin and eosin. (A) CONTROL ovary showed mostly healthy follicles with no sign of GC damage. (B) Ovary treated with LOW-DOC showed several degenerating follicles, with pyknotic GCs (black arrowhead) and damaged follicles (black arrow). (**C**–**F**) Immunohistochemical localization of mouse vasa homologue (MVH: red) and cleaved caspase 3 (CC3: green), illustrating the degree and site of apoptosis. (C) CONTROL ovary exhibiting many MVH-positive oocytes but with little sign of CC3-positive apoptotic cells. (D–F) DOC-treated sections displayed marked apoptosis staining, with CC3 signal evident in many GCs (white arrowhead) and stroma (white arrow). Sections were counterstained with DAPI (blue). Insets represent magnified images of respective framed areas. (**G** and **H**) Analysis of immunohistochemistry for CC3. DOC-treated ovaries showed significantly increased expression of CC3 whether analysed (G) manually or (H) semi-automatically. (G) Percentage of follicles containing CC3-positive cells. (H) Area of CC3-positive cells relative to area of section. Total of ovaries used in three independent experiments: CONTROL= 5, LOW-DOC = 5, MID-DOC = 5 and HIGH-DOC = 4. Kruskal–Wallis followed by Dunn *post hoc* tests were applied. Data are mean ± SEM. *P* < 0.05 (*) for DOC versus CONTROL.

In order to analyse which follicle cell type was the principal target of DOC damage, the percentage of follicles with unhealthy oocyte only, GCs only or both oocyte and GCs (OO + GCs) was determined (Fig. 2). When the total follicle population was considered, there was a significant increase in the percentage of follicles with unhealthy oocytes relative to CONTROL only in the HIGH-DOC group (Fig. 2A-i). In contrast, GCs were markedly affected by DOC exposure even at the lowest drug concentration: the percentage of unhealthy follicles because of unhealthy GCs relative to the total number of follicles significantly increased at all doses tested, compared with no follicles classified as unhealthy due to poor GCs in the CONTROL group (Fig. 2A-ii). The percentage of follicles categorized as unhealthy due to unhealthy oocytes and GCs (OO + GCs) was also significantly greater in the MID- and HIGH-DOC groups compared with CONTROL (Fig. 2A-iii). When each stage of follicle development was analysed separately, primordial follicles showed no signs of damage in response to drug treatment over that seen in CONTROL primordial follicles (Fig. 2B-i, B-ii, B-iii). In contrast, growing (transitional and primary) follicles were in most cases significantly affected by DOC treatment, exhibiting poor GC, or OO+GC health, even if not in a dose–response manner (Fig. 2B-ii, B-iii). Overall, results indicate that the primary cell type affected by DOC exposure is the GCs of growing follicles, with significant increase in degeneration even at the lowest concentration. Growing follicles of ovaries exposed to LOW-DOC dose showed signs of pyknosis primarily localized to the GCs (Fig. 3A–D). 

### Docetaxel induces apoptosis of ovarian stromal and follicular cells

**Figure 4:**
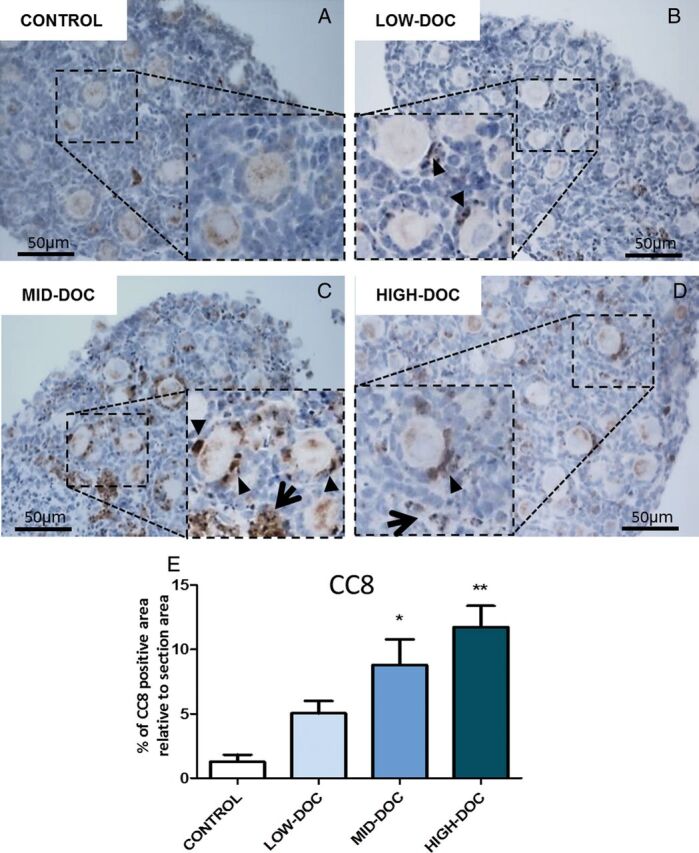
Docetaxel activates caspase 8. (**A**–**D**) Representative photomicrographs of mouse ovarian sections processed using immunohistochemistry to localize cleaved caspase 8 (CC8: brown), to illustrate the degree of apoptosis. (A) CONTROL ovary exhibiting little sign of CC8-positive apoptotic cells. (B–D) DOC-treated sections displaying marked apoptosis staining, with CC8 signal evident in many GCs (arrowhead) and stromal cells (arrow). Sections were counterstained with Haematoxylin (blue). Insets represent magnified images of respective framed areas. (**E**) Semi-automated analysis of immunohistochemistry. Exposure to DOC increased expression of CC8 relative to section area with the MID- and HIGH-DOC doses. Total of ovaries used in three independent experiments: CONTROL = 4, LOW-DOC = 4, MID-DOC = 6 and HIGH-DOC = 6. Kruskal–Wallis followed by Dunn *post hoc* tests were applied. Data are mean ± SEM. *P* < 0.05 (*), *P* < 0.01 (**) for DOC versus CONTROL.

**Figure 5:**
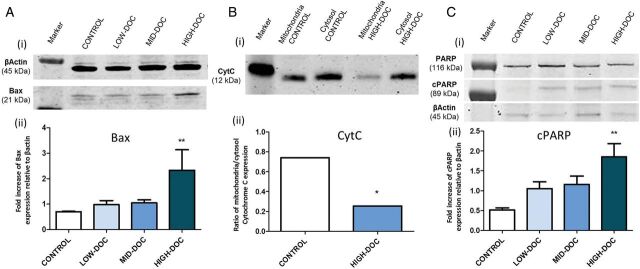
Docetaxel exposure increases Bax expression, releases Cytochrome C into the cytosol and cleaves poly(ADP-ribose) polymerase (PARP) in mice. Ai, Bi and Ci: Representative images of western blotting of whole ovary proteins, for (**A**) Bax, (**B**) Cytochrome C and (**C**) cleaved PARP (cPARP). Aii, Bii and Cii: Densitometric analyses of proteins, expressed as relative to that of β-actin (Aii, Cii) or as a ratio of mitochondrial: cytosol expression (Bii). Bax and cPARP expression increased significantly only when ovaries were exposed to HIGH-DOC. HIGH-DOC treatment also induced release of Cytochrome C from the mitochondria into the cytosol. For Bax, a total of six ovaries were used for each treatment group in three independent experiments and Kruskal–Wallis followed by Dunn *post hoc* tests were applied. For CytC, a total of 10 ovaries were used for each treatment group in five independent experiments and a paired *t*-test was used. For cPARP, a total of 10 ovaries were used for each treatment group in five independent experiments and Kruskal–Wallis followed by Dunn *post hoc* tests were applied. Data are mean ± SEM. *P* < 0.01 (**) for DOC versus CONTROL.

The ability of DOC to induce activation of the intracellular apoptotic pathway in the mouse ovary was tested by analysing the expression of the following programmed cell death markers: CC3 (*n* = 5 each for CONTROL and LOW- and MID-DOC, *n* = 4 for HIGH-DOC) and CC8 (*n* = 4 for CONTROL and LOW-DOC, *n* = 6 for MID- and HIGH-DOC), both examined using IHC. In addition, western blotting was used to assess expression of Bax (*n* = 3 for all groups), release of Cytochrome C (CytC) from mitochondria into the cytosol (*n* = 5 for both CONTROL and HIGH-DOC groups) and cleavage of PARP (cPARP) (*n* = 5 for all groups). CC3 expression was analysed both by manual counting and also using a more automated system. Firstly, for manual counting, the number of follicles with oocyte and/or GCs that were CC3-positive was recorded and expressed relative to the number of follicles in the section, with follicle counts determined by numbers of MVH-expressing oocytes (Fig. 3C–F). DOC treatment significantly increased the proportion of CC3-positive apoptotic follicles relative to CONTROL (Fig. 3G). Secondly, for the semi-automated system, the CC3-expressing area of somatic (granulosa and stromal) cells within a section was measured and analysed relative to total section area; as with the manual counting, the area of apoptosis increased in all DOC-treated groups (Fig. 3H). The degree of activation of programmed cell death of follicles detected by CC3 expression thus reflected the degree of damage observed by morphological observation after DOC treatment. Furthermore, the level of CC3 expression within the follicles of DOC-treated ovaries was comparable with its level in the somatic cells: in both cases CC3 increased 8–11 fold versus CONTROL. It is noteworthy that the matching of the outcomes in both systems confirms the reliability of this semi-automated system and for this reason, only the latter system was chosen to analyse CC8 expression (Fig. 4). All treated groups showed an increase in CC8 levels in comparison with untreated tissue (Fig. 4A–D), with the increase following a dose–response pattern and significant at MID- and HIGH-DOC doses (Fig. 4E). In order to further explore the DOC-induced apoptotic pathway, western blotting analysis for Bax, CytC and cPARP was performed (Fig. 5). Bax expression significantly increased 3.3-fold (relative to β-actin) over CONTROL in the HIGH-DOC group (Fig. 5A-ii). Exposure to HIGH-DOC also induced release of CytC from mitochondria into the cytosol, leading to a 3-fold decrease in the ratio of mitochondrial:cytosol CytC (Fig. 5B-ii). PARP cleavage showed a significant increase of 3.6-fold (relative to expression of β-actin) only in the HIGH-DOC group (Fig. 5C-ii). 

### T3 does not protect ovarian follicles from Docetaxel-induced damage

**Figure 6:**
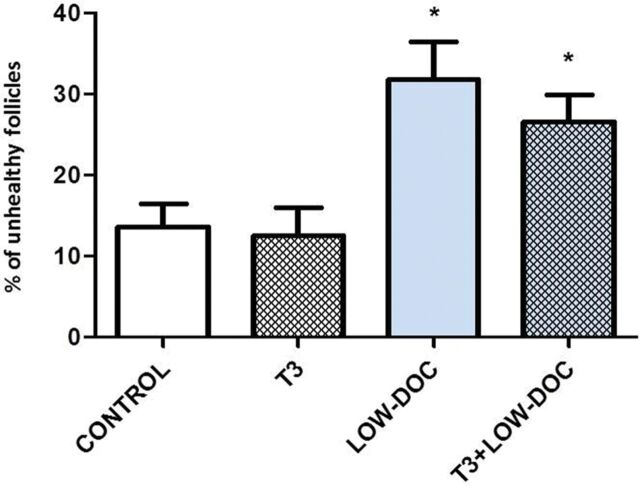
Thyroid hormone (T3) does not mitigate DOC damage on mouse ovary. Percentage of unhealthy follicles in ovary after culture: the significant increase in the percentage of follicles that were unhealthy following exposure to LOW-DOC did not decrease when the ovaries were also exposed to T3. Total of ovaries used per each group in four independent experiments: CONTROL = 7, T3 = 8, LOW-DOC = 6 and T3+LOW-DOC = 10. Kruskal–Wallis followed by Dunn *post hoc* tests were applied. Data are mean ± SEM. Asterisks show statistical significance of DOC and T3+DOC treatments relative to CONTROL group. *P* < 0.05 (*).

In order to test whether T3 plays a putative protective role against DOC damage, medium was supplemented with T3 in the presence (T3 + LOW-DOC, *n* = 10) or absence (T3, *n* = 8) of LOW-DOC, with data compared with CONTROL group (*n* = 7) or LOW-DOC alone (*n* = 6). T3 alone did not affect normal ovarian morphology, with the percentage of unhealthy follicles similar to the CONTROL group. As in the previous experiment (Fig. 1B-i), LOW-DOC significantly increased the percentage of follicles classified as unhealthy (Fig. 6). A similar degree of damage was obtained when LOW-DOC and T3 were added together (Fig. 6). There is, therefore, no indication that T3 is able to protect ovarian follicles from DOC-induced damage at the concentrations used here. 

## Discussion

The potential to design protective agents and strategies to reduce ovarian toxicity from chemotherapy regimens requires detailed understanding of the affected cells and pathways involved. Using an *in vitro* mouse model we have shown that DOC directly impairs the early stages of ovarian follicle development, but does not directly affect the resting pool of primordial follicles. Furthermore, we demonstrated that GCs of growing follicles are the first cellular target of DOC-induced follicular damage, indicating that oocyte damage is indirect, and that DOC induces damage of somatic cells following a mitochondrial-dependent apoptotic pathway. No protective effect of T3 was found.

Results here showed that DOC induces loss of follicles in the very early stages of growth even at concentrations of DOC (0.1 µM), lower than that found in the plasma of cancer patients (Slaviero *et al.*, 2004; Baker *et al.*, 2004; Brunsvig *et al.*, 2007). This effect must be due either to decreasing numbers of follicles reaching the primary stage or increasing numbers dying soon after they reach the primary follicle stage. Moreover, exposure to these low concentrations of DOC severely impaired follicle health, with abnormal morphology seen in ∼30% of transitional follicles and >80% of primary follicles. This scenario became even more severe at drug concentrations in the higher range of plasma levels to be found in DOC-treated patients.

Several groups have used *in vivo* and *in vitro* rodent models to test the effect of ovotoxic compounds which are used in human medicine (for example, Petrillo *et al.*, 2011; Maiani *et al.*, 2012). The culture system used in the present study has been previously used in our laboratory to test the damage caused to the ovarian follicle population by other chemotherapeutic agents, including examination of the chemotherapy drug cisplatin that is considered to have moderate clinical ovotoxicity (Morgan *et al.*, 2013): *in vitro* exposure of ovaries to clinically relevant doses of cisplatin induced a similar degree of damage to that observed in the present study, in which ovaries were exposed to clinically relevant doses of DOC. Bearing in mind the need for caution in extrapolating results from an *in vitro* animal model to consideration of the effects of DOC on human patients, this does together indicate that chemotherapeutic doses of DOC may well induce moderate levels of ovarian toxicity: certainly, further examination of the effects of DOC on the fertility of younger female patients is warranted.

The limited information about the impact of DOC on patient fertility that is available largely refers to the length of temporary, or time until permanent, amenorrhoea, which are not good indicators of partial ovarian damage (Berliere *et al.*, 2008; Zhou *et al.*, 2010). Furthermore, within the studies examining the degree of amenorrhoea following DOC treatment, some have suggested a marked effect of DOC (Martin *et al.*, 2005; Han *et al.*, 2009), while others have not (Minisini *et al.*, 2009; Zhou *et al.*, 2010), although a more detailed analysis indicated that taxane administration in combination with other chemotherapy agents does add to the treatment's ovarian toxicity (Anderson *et al.*, 2006). To the best of our knowledge, no data are available from animal models to determine detrimental effects of DOC on fertility. There are some *in vivo* studies testing the effect of the first generation taxane, paclitaxel, on primordial follicles in adult mice, but these are contradictory. Injection of 7.5 mg/kg of paclitaxel (considered a high dose) in a single or repeated administration, decreased primordial follicle count 1 week after drug exposure (Gucer *et al.*, 2001; Yucebilgin *et al.*, 2004), while in contrast, another *in vivo* study used repeated administration of paclitaxel (5 mg/kg) on adult rats, but found no reduction of follicle counts at any pre-antral development stage, with only antral follicles affected (Tarumi *et al.*, 2009). Mature oocytes exposed to paclitaxel undergo a delay of nuclear maturation and defective spindle formation, leading to aneuploidy (Mailhes *et al.*, 1999).

Women undergoing chemotherapy usually receive several treatments over a period of months. As human ovarian follicles take ∼6 months to develop from the dormant state to the fully grown ovulatory follicles (McGee and Hsueh, 2000), each treatment will affect a range of follicles at different developmental stages. The major concern about ovarian damage is if it markedly affects the ovarian reserve of resting primordial follicles, as this can lead to POI. Primordial follicle loss could result from a direct effect of damage to the primordial pool, or could be an indirect effect, where the decrease in primordial follicle numbers is through an increased rate of growth initiation due to a major loss of growing follicles, a process termed the burnout model (Roness *et al.*, 2013). Although some authors consider mature follicles to be more sensitive to chemotherapy than small follicles (Nicosia *et al.*, 1985; Gucer *et al.*, 2001), damage specifically to the small number of follicles that are at the late antral stage at any one time point should have, at most, a minor impact on long-term fertility. The present results demonstrated that immature, early growing follicles are severely affected by DOC, in both their number and quality, even at low DOC concentrations. Since early growing follicles are present in large numbers, marked damage to this follicle pool could well result in follicular burnout (Kalich-Philosoph *et al.*, 2013; Morgan *et al.*, 2013). The burnout model is also in accordance with the observations of others using high doses of paclitaxel on mice *in vivo* (Gucer *et al.*, 2001; Yucebilgin *et al.*, 2004). In the present study, primordial follicle number did not decrease significantly, but a negative trend was observed at the highest DOC concentration. This may reflect the particular characteristic of the present model, as the primordial follicle pool in mouse is not completely formed at birth and the short duration of the experiment may not allow full elaboration of secondary effects.

Given that the oocyte and somatic cells of the follicle are mutually interdependent, damage to the latter would inevitably affect the health of the oocyte as well. In addition, the loss of stromal integrity could lead to subsequent follicle damage. The present study showed that DOC severely affects early growing follicles, initially damaging GCs, with oocyte damage becoming apparent only after GCs were compromised. Our data also suggest that DOC negatively impacts on stromal components of the ovary, as has been seen from other chemotherapy drugs. A timeline study of the damage produced by doxorubicin, an anthracycline agent used in cancer treatment, revealed that, in the mouse, stromal tissue is the first target, probably due to its contact with the circulatory system (Roti Roti *et al.*, 2012). Furthermore, human ovarian cortex of cancer patients showed damage to blood vessels and fibrosis, suggesting that the loss of stroma integrity could lead to follicle damage (Meirow *et al.*, 2007). The explanation for the DOC-induced damage probably lies in the principal mechanism of action of this group of chemotherapeutic drugs: taxanes promote microtubule assembly and inhibit their polymerization, leading to mitotic arrest and apoptosis. Mitotically active cells, such as GCs and stromal cells, are, for that reason, more likely to be vulnerable to DOC damage than oocytes. Oocyte death would follow as a downstream consequence of GC damage and loss of oocyte-GC connections (Thomson *et al.*, 2010). This DOC mechanism of action may also explain the absence of a dose–response pattern of damage in much of the data presented here: the plateau of damage is perhaps already reached at the lowest concentration able to perturb the cell cycle in dividing GCs within activated-early growing follicles and stroma cells at the time of DOC exposure. Thus, if the ovary was exposed to repeated doses rather than higher doses, this would affect an increasing number of cells subsequently entering into the G2/M phase, and could well lead to more wide spread damage. Similarly, a cytometric study using a bladder cancer cell line (HT1376, American Type Culture Collection line) failed to find a dose–response relationship after 1 h of DOC treatment, while 24 h treatment induced dose-dependent cytostatis (Fabbri *et al.*, 2006).

**Figure 7:**
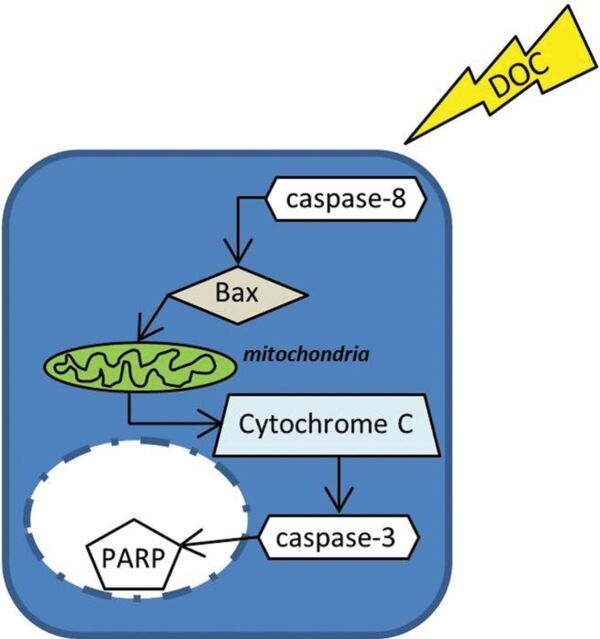
Type II, mitochondrial-dependent pathway by which DOC induced apoptosis in ovarian granulosa cells. Exposure of granulosa cells to DOC induces cleavage of caspase 8, leading to an up-regulation of Bax. This in turn activates the intrinsic apoptotic pathway, involving a mitochondrial-dependent cascade in which the mitochondrial membrane becomes increasingly permeable, leading to increased movement of Cytochrome C into the cytosol. Cytochrome C then activates downstream effector caspases, such as caspase 3, which in turn leads to cleavage of PARP, inactivating its ability to support DNA repair.

Here, we show that DOC activates the intrinsic, mitochondrial-dependent apoptotic pathway in the somatic compartment of the ovary, inducing expression of key cell death markers (for schematic, see Fig. 7). Pro-caspase 8 is thought to be the first intracellular cysteine protease activated upon binding of a cell death receptor (Manabe *et al.*, 2004). GCs then activate the intrinsic, type II mitochondrial-dependent apoptotic cascade, subsequently interacting with pro-apoptotic Bcl-2 family members such as Bax (Sai *et al.*, 2012). Bax is a pro-apoptotic protein that plays a principal role in the mitochondria involvement in cell death. It undergoes conformational changes, integrating into the mitochondrial membrane and hence disrupting its membrane potential. That in turn induces an increase in the permeability of the mitochondrial membrane, with a consequent release of cytochrome C from mitochondria into the cytosol (Liu *et al.*, 2006; Zhao *et al.*, 2010; Khan *et al.*, 2014). Cytochrome C activates the downstream caspases, including effector caspases such as caspase 3, which in turn leads to cleavage of the nuclear substrate PARP, inactivating its ability to support DNA repair. 

In the longer term, it may be possible to develop protective measures against the gonadotoxic effects of chemotherapeutic agents. The proposed protection exerted by thyroid hormone (T3) was investigated here. Thyroid hormones are involved in the development and functionality of the reproductive system, with both hypo- and hyperthyroidism associated with subfertility (Stavreus-Evers, 2012). T3 has been proposed to prevent paclitaxel-induced apoptosis in a rat GC line (Verga Falzacappa *et al.*, 2012). The present studies show that the same dose of T3 was unable to prevent the ovarian damage induced by a low dose of DOC. DOC and paclitaxel do differ: DOC binds tubulin with higher affinity than paclitaxel and is capable of direct induction of apoptosis via bcl-2 phosphorylation (Gligorov and Lotz, 2004). Nonetheless, the discrepancy between our data and the results of Verga Falzacappa *et al.* (2012) could instead be due to the markedly different experimental paradigms used: we suggest that the organ culture system utilized here, where follicles are maintained intact and able to develop intact, as *in vivo*, is much more physiological than a GC line. While DOC dose was representative of the lowest doses found in serum samples of treated cancer patients, the T3 concentration used here (10^−7^ M) and in Verga Falzacappa *et al.* (2012) is almost 30 times higher than the serum concentration of T3 in healthy people and 8 times that in patients with thyrotoxicosis (Sterling *et al.*, 1969).

In conclusion, the present study in mice shows clear evidence of ovarian toxicity of DOC at clinically relevant concentrations. The model used does not allow analysis of effects on later stages of follicle growth, but demonstrates effects on the stages highly relevant to long-term fertility, namely that of early growing follicles. DOC was demonstrated here to cause loss of primary follicles, but with no evidence of direct effects on primordial follicles. Primordial follicles could however be the site of secondary effects, with increased activation and thus premature depletion.

## Authors' roles

F.L., R.A.A. and N.S. designed experiments; F.L. and R.S. performed experiments; F.L., R.A.A. and N.S. interpreted data; F.L. and N.S. wrote the manuscript; R.S. and R.A.A. critically revised the manuscript.

## Funding

This work was supported by Medical Research Grant (MRC) grant G1002118. Funding to pay the Open Access publication charges for this article was provided by the Univeristy of Edinburgh's Institutional publication fund.
